# Subtotal (segment II–VIII) hepatectomy for bilateral diffuse hepatolithiasis with compensatory caudate lobe hypertrophy: a report of two cases

**DOI:** 10.1186/s12876-020-01503-9

**Published:** 2020-10-20

**Authors:** Wei Wang, ZiJie Zhang, Jian Wang

**Affiliations:** 1 Department of Hepatobiliary and Pancreatic Surgery, Shanghai Jiao Tong University Affiliated Sixth People’s Hospital, Shanghai, 200233 China; 2 Department of Biliary-Pancreatic Surgery, Renji Hospital, Shanghai Jiao Tong University School of Medicine, Shanghai, 200127 China

**Keywords:** Hepatolithiasis, Atrophy–hypertrophy complex, Caudate lobe, Subtotal hepatectomy

## Abstract

**Background:**

Hepatolithiasis often leads to atrophy–hypertrophy complex due to bile duct obstruction, inflammation or infection in the affected liver segments and compensatory response in the normal segments. In severe bilateral diffuse cases, main liver can all be atrophic, leaving the caudate lobe to be extremely hypertrophic. Subtotal (segment II–VIII) hepatectomy can be an option in selected patients under such circumstances. Since rare cases have been reported, our study aims to highlight the preoperative evaluation and key points of this procedure.

**Case presentation:**

Two patients with primary and secondary bilateral diffuse hepatolithiasis, respectively, were enrolled in this case series. The atrophy of the left and right liver with an exceeding hypertrophy of the caudate lobe were observed. Since the liver anatomy had completely been changed, contrast computed tomography, magnetic resonance imaging combined with 3D liver reconstruction were employed for comprehensive evaluation and pre-operational planning. The patients underwent standard subtotal (segment II–VIII) hepatectomy. During operation, the hepatoduodenal ligament around porta hepatis was dissected firstly to expose the hepatic artery, portal vein, bile duct and their branches successively. And then the vessels and bile duct to caudate lobe were preserved safely through cutting off the left and right hepatic artery, portal vein and bile duct at a safe point distal to the origin of the branches to caudate lobe. Operation time was 300 min and 360 min, respectively. Blood loss was 200 ml and 300 ml. No evidence of liver dysfunction, hepatolithiasis relapse or cholangitis was observed during the follow-up of 12 and 26 months.

**Conclusions:**

Subtotal (segment II–VIII) hepatectomy may be one of several treatments possible in selected patients with compensatory caudate lobe hypertrophy caused by bilateral diffuse hepatolithiasis.

## Background

Hepatolithiasis is a complicated and challenging disease characterized by formation of stones in the intrahepatic bile duct with the chronic but progressive nature. Incidence and etiology of hepatolithiasis varies dramatically. In east Asia, this prevalent disease is often primary with a reported incidence between 3.1% and 21.2% [1]. However, in western countries, hepatolithiasis with the incidence of 1% approximately tends to be secondary to cholestasis (e.g. Caroli Disease), biliary stricture (e.g. common bile duct injury) or cholangitis (e.g. primary sclerosing cholangitis or infection). It’s noteworthy that there have been emerging cases of hepatolithiasis in north America since immigrants from epidemic regions of the world increased enormously [2, 3].

Manifestation of hepatolithiasis ranges from mild chronic abdominal pains to severe refractory cholangitis. Stones, infections, inflammations and strictures promote the exacerbation of the disease mutually, leading to the atrophy in the affected liver segments and compensatory hypertrophy in the uninvolved parts [4]. This atrophy–hypertrophy complex (AHC) along with changes of anatomic configuration like portal triads may be a thorny issue during surgical intervention. Under extremely rare circumstances, the main liver (Couinaud’s segment II–VIII) can be almost totally atrophic while the caudate lobe (Couinaud’s segment I) has been exceedingly hypertrophic. Subtotal (segment II–VIII) hepatectomy instead of liver transplant has scarcely been reported in the literature to treat bilateral diffuse hepatolithiasis [5, 6]. The introduction of our experience aims to describe the precise preoperative evaluation and challenging procedure details.

## Case presentation

### Case 1

**Fig. 1:**
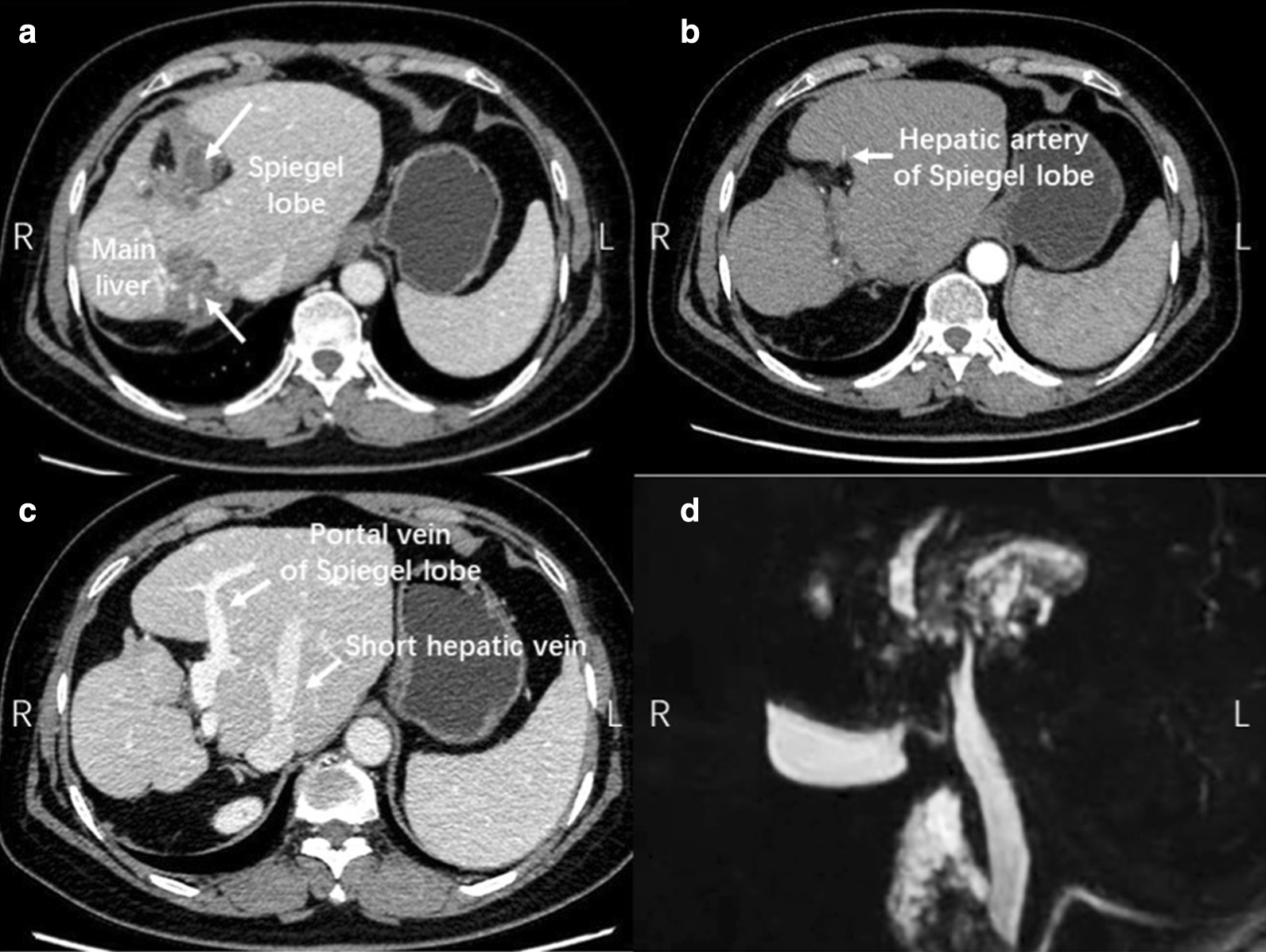
Imaging assessment of case 1. **a** CT image showed hepatolithiasis in the right liver and left liver (arrow). **b** Contrast CT image showed hepatic artery of Spiegel lobe. **c** Contrast CT image showed hepatic vein and portal vein of Spiegel lobe of the caudate lobe. **d** MRCP showed no stricture in extrahepatic bile duct

A 58-year-old female was admitted due to discomfort in the right upper quadrant of abdomen for over 20 years. Physical examination showed no abnormality. Liver function tests were normal (Child–Pugh A grade). Contrast-enhanced CT and MRI of liver demonstrated bilateral diffuse hepatolithiasis and atrophy in left and right lobes with exceeding hypertrophy of caudate lobe. There was no evidence of stricture in extrahepatic bile duct (Fig. 1). The volume of hypertrophic caudate lobe was 68.4% according to 3-dimentional reconstruction of CT scan.

### Case 2

**Fig. 2:**
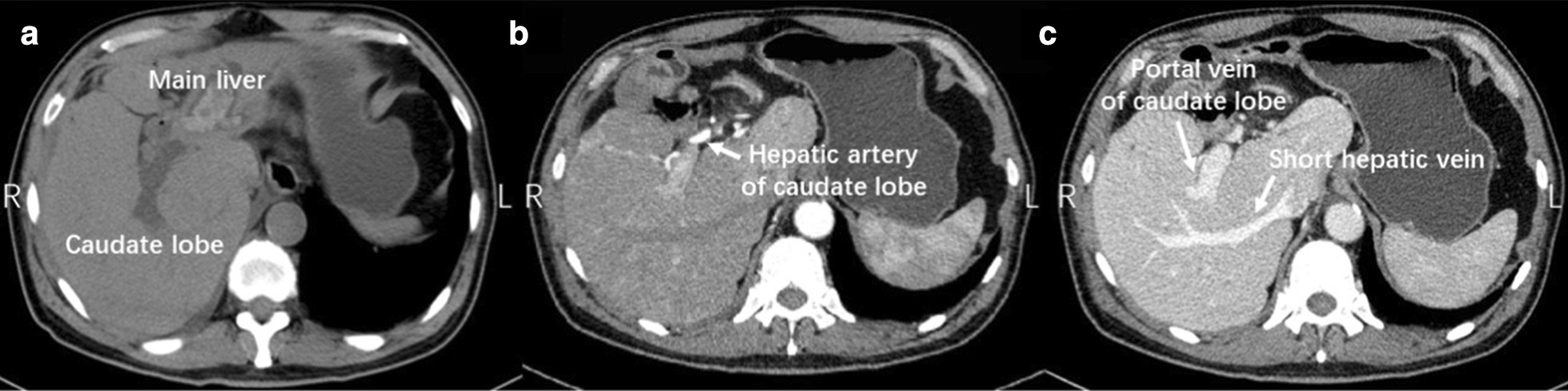
Imaging assessment of case 2. **a** CT image showed hypertrophic caudate lobe and hepatolithiasis in the main liver. **b** Contrast CT image showed hepatic artery of the caudate lobe. **c** Contrast CT image showed hepatic vein and portal vein of the caudate lobe

A 63-year-old male presented with a 17-year history of right upper abdominal pain and intermittent fever. Past medical history included a choledochocystectomy with bilioenterostomy 24 years ago. Mild jaundice was observed. Liver function tests showed a serum total bilirubin of 51.7 mmol/L, alkaline phosphatase of 142 μmol/L, γ-GT of 245 μmol/L, indicating biliary obstruction (Child–Pugh A grade). The results of contrast-enhanced CT and MRI were similar to case 1 and RLV was 65.8% (Fig. 2).

#### Atrophy–hypertrophy complex

Contrast CT was used for the evaluation of AHC. Normally, the short hepatic veins draining the caudate lobe are too tiny to be seen on CT images. But under the condition of exceeding caudate lobe hypertrophy, its short hepatic veins caudal to the hepatic veins of the main liver become thick and usually run in the middle of hypertrophic caudate lobe, which could be easily identified by CT images. Moreover, the location of these short hepatic veins could indicate which part of the caudate lobe is hypertrophic. If these short hepatic veins are on the left side of IVC (inferior vena cava), it indicates the hypertrophy of the Spiegel lobe. On this occasion, the atrophic main liver will rotate counterclockwise towards right side, while the hepatic hilum might rotate to the dorsal of the abdominal cavity, resulting in difficulty to dissect the hilum (Case 1, Fig. 1). On the contrary, if these short hepatic veins located on the right side of IVC, the hypertrophy of the paracaval portion and caudate processus would push the main liver clockwise rotating towards left side (Case 2, Fig. 2), resulting the hepatic hilum become more superficial.

#### Configuration of the vasculatures

**Fig. 3.**
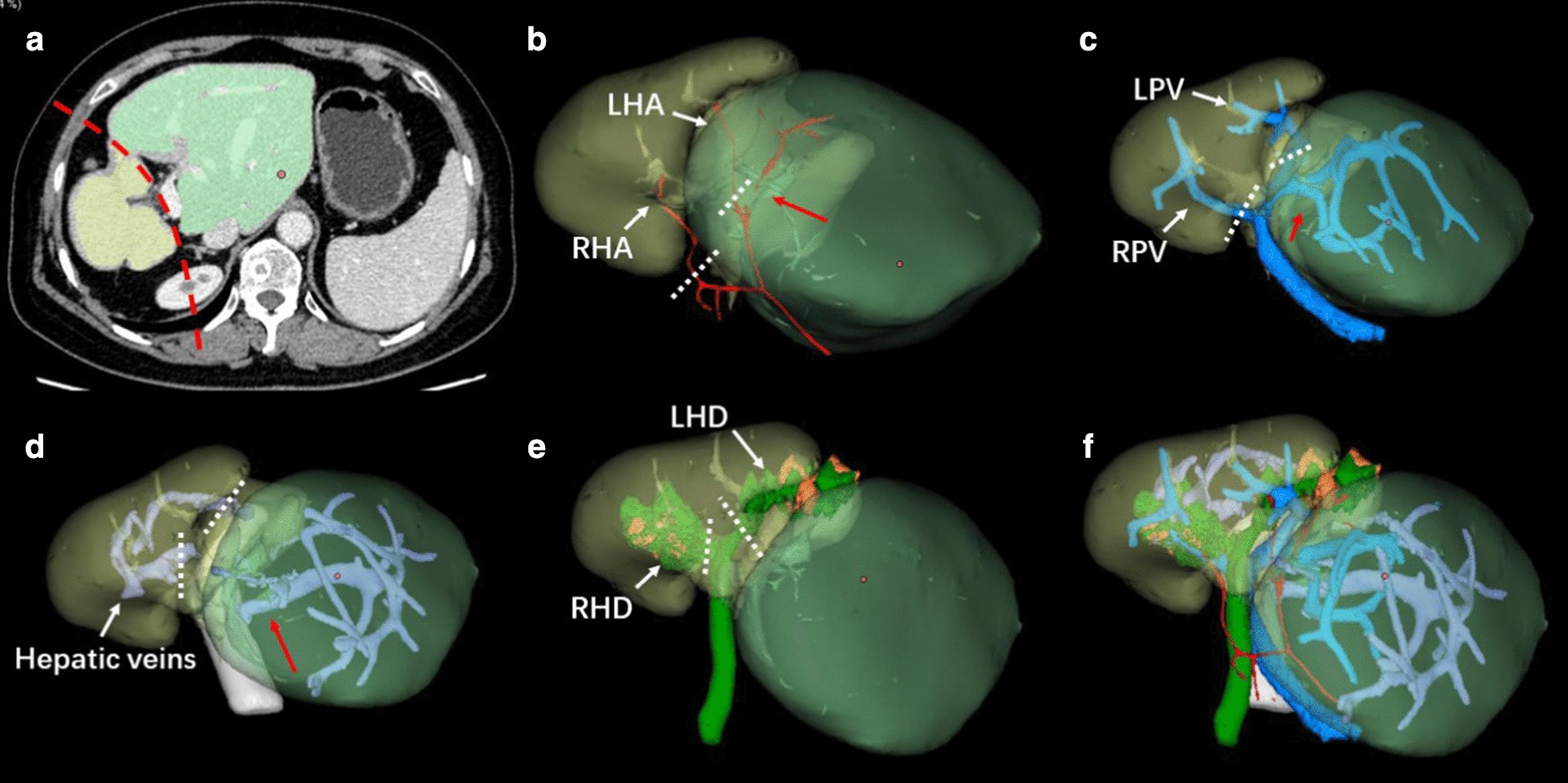
3D liver reconstruction of case 1. **a** Reflection of 3D reconstruction in CT image. Green part: hypertrophic caudate lobe; Yellow part: atrophic main liver; Dashed red line: boundary of caudate lobe and main liver. **b** Reconstruction of hepatic arteries. Dashed white line: transection of hepatic arteries; Solid red arrow: hepatic artery of caudate lobe. **c** Reconstruction of portal veins. Dashed white line: transection of portal veins; Solid red arrow: portal vein of caudate lobe. **d** Reconstruction of hepatic veins. Dashed white line: transection of hepatic veins; Solid red arrow: short hepatic veins. **e** Reconstruction of bile ducts. LHD and RHD were filled with stones. Dashed white line: transection of hepatic ducts. **f** Configuration of the vasculatures. *LHA* left hepatic artery, *RHA* right hepatic artery, *LPV* left portal vein, *RPV* right portal vein, *LHD* left hepatic duct, *RHD* right hepatic duct

**Fig. 4.**
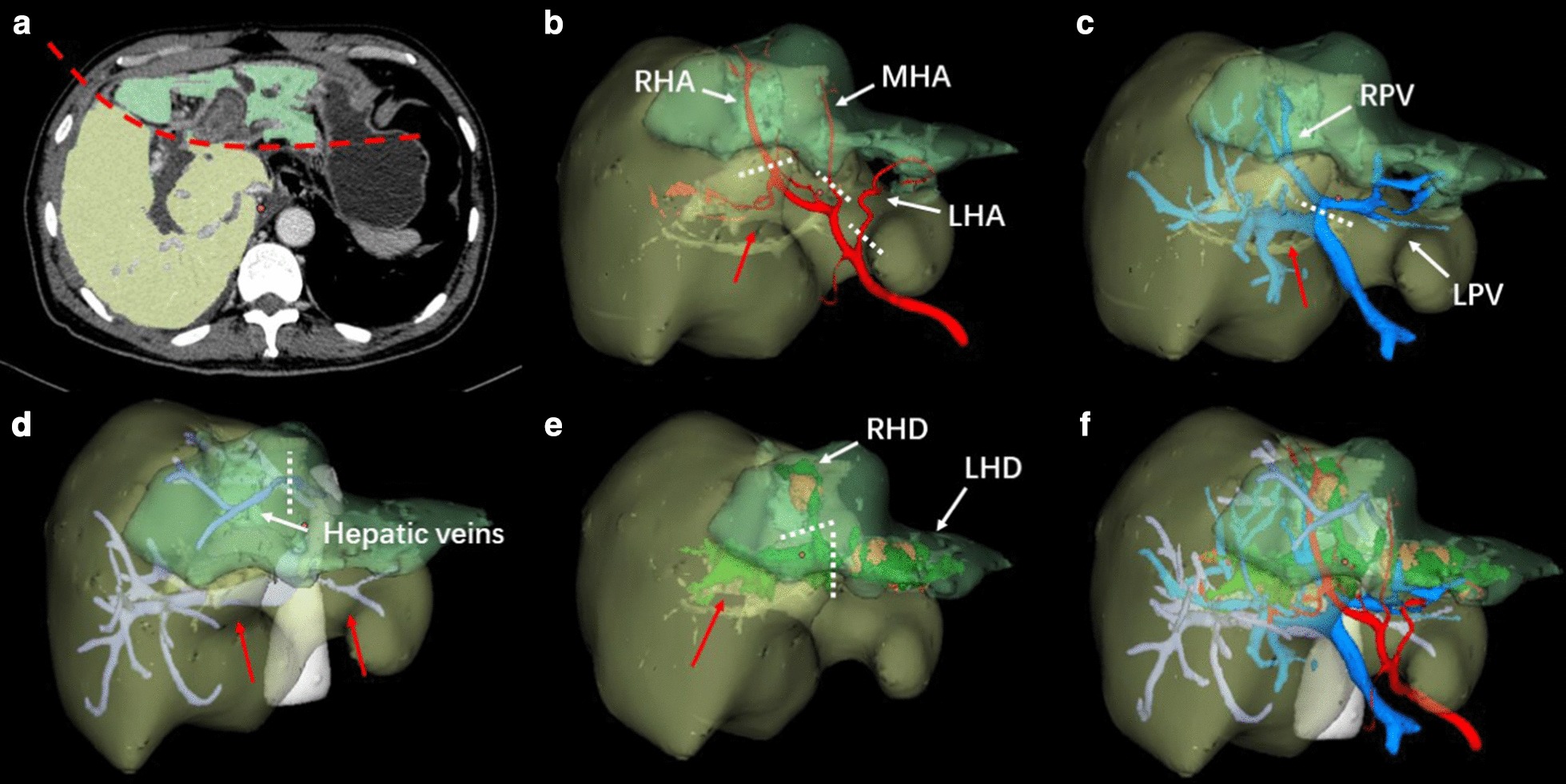
3D liver reconstruction of case 2. **a** Reflection of 3D reconstruction in CT image. Yellow part: hypertrophic caudate lobe; Green part: atrophic main liver; dashed red line: boundary of caudate lobe and main liver. **b** Reconstruction of hepatic arteries. Dashed white line: transection of hepatic arteries; Solid red arrow: hepatic artery of caudate lobe. **c** Reconstruction of hepatic veins. Dashed white line: transection of portal veins; Solid red arrow: portal vein of caudate lobe. **d** Reconstruction of hepatic veins. Dashed white line: transection of hepatic veins; Solid red arrow: short hepatic veins. **e** Reconstruction of bile ducts. LHD and RHD were filled with stones. Dashed white line: transection of hepatic ducts; Solid red arrow: bile duct of caudate lobe. **f** Configuration of the vasculatures. *LHA* left hepatic artery, *MHA* middle hepatic artery, *RHA* right hepatic artery, *LPV* left portal vein, *RPV* right portal vein, *LHD* left hepatic duct, *RHD* right hepatic duct

Contrast CT along with 3D liver reconstruction was employed to evaluate configuration or variation of the vasculature system, especially the blood supply or bile drainage of caudate lobe. In case 1, the artery and portal vein of caudate lobe that originate from left hepatic artery (LHA) and left portal vein (LPV) respectively were prone to be observed, but it was difficult to identify the undilated bile duct of caudate lobe in 3D images (Fig. 3). In case 2, 3D images showed that the artery, portal vein and bile duct of caudate lobe originated from right hepatic artery (RHA), main portal vein and confluence of right and left hepatic ducts respectively (Fig. 4).

#### Treatment

**Fig. 5:**
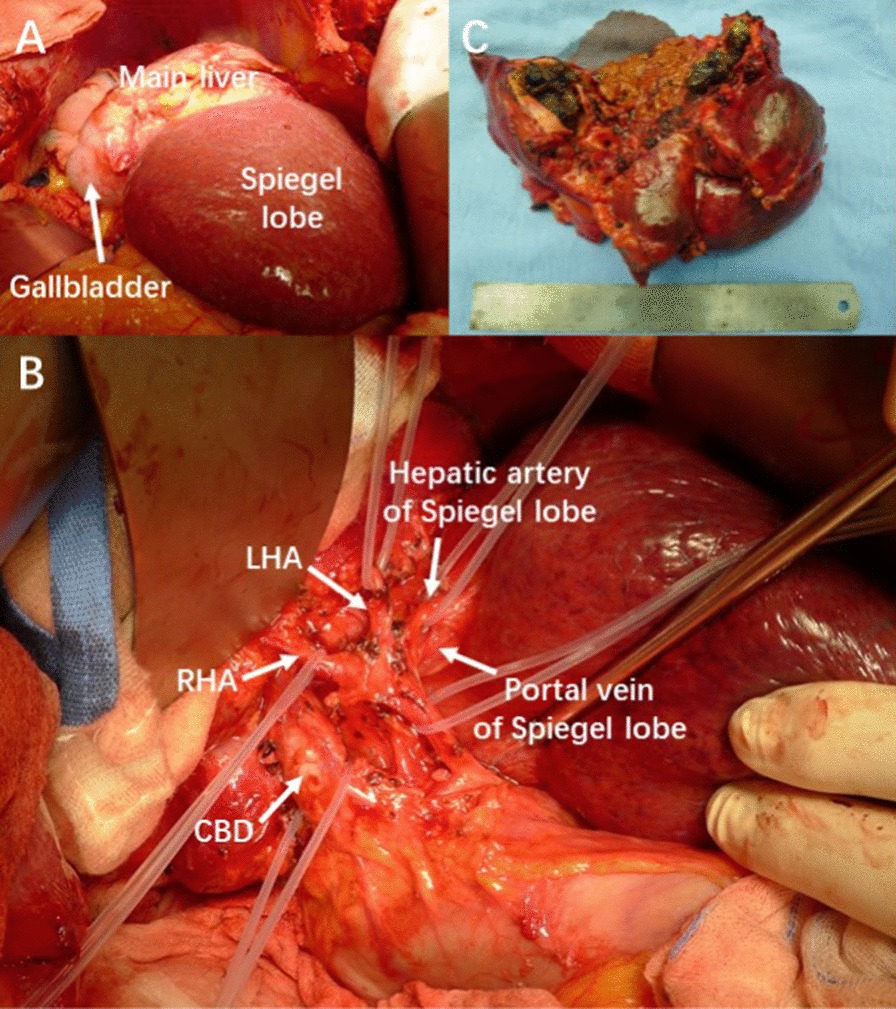
Intraoperative finding of case 1. **a** The main liver was atrophic with Spiegel lobe hypertrophic. **b** The hepatic artery, portal vein and bile duct were exposed after hepatoduodenal ligament is dissected. **c** Bilateral liver lobes were filled with diffuse pigment stones. *LHA* left hepatic artery, *RHA* right hepatic artery, *CBD* common bile duct

Operation was started with a right subcostal reversed L-shaped incision. Intraoperative exploration: In case 1, the caudate lobe was extremely hypertrophic with normal texture, while the segment II- VIII were atrophic and contained a large amount of bile pigment stones (Fig. 5). There were severe adhesions between atrophic liver and diaphragm or IVC. The diameter of common bile duct was 1.5 cm. The distal bile duct was unobstructed with normal Oddi sphincter and the opening orifice of bile duct for caudate lobe was not stenosed. So caudate lobe-sparing subtotal hepatectomy and choledochoplasty with T tube drainage were performed (Additional file 1: Video 1).

**Fig. 6:**
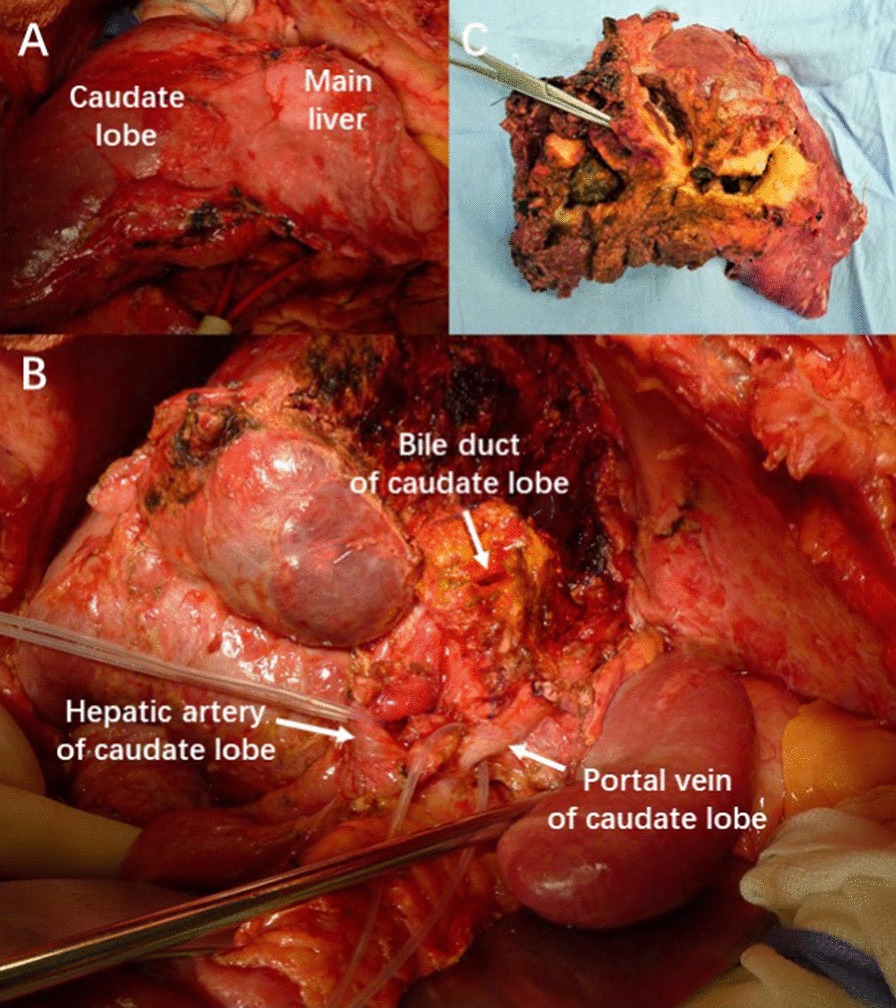
Intraoperative finding of case 2. **a** The main liver was atrophic with caudate lobe hypertrophic. **b** The hepatic artery, portal vein and bile duct were exposed after hepatoduodenal ligament is dissected. **c** Bilateral liver lobes were filled with diffuse pigment stones

In case 2, there were severe adhesions in the upper abdomen. The previous surgical procedure was hepaticojejunostomy. The segment II–VIII were atrophic with the left and right intrahepatic bile ducts filled with stones and pus (Fig. 6). The caudate lobe was hypertrophy with normal texture. So caudate lobe-sparing subtotal hepatectomy and hepaticojejunostomy were carried out.

#### Mobilization of the main liver

The liver was mobilized via dividing the contractual and dislocated falciform, triangular and coronary ligaments. By the use of low-power electric knife, meticulous layer-by-layer separation of the adhesions was performed to avoid deflecting to either diaphragm or liver parenchyma. Especially, in case 1 the Makuuchi ligament was divided to well expose IVC and transect the short hepatic vein from the right liver, while venous drainage of the caudate lobe was preserved.

#### Hepatic hilum exposure

The hepatic hilum was well exposed after dissection of hepatoduodenal ligament in case 1 or the previous intestinal loop for biliary-intestinal anastomosis in case 2. Cholecystectomy was performed after dissecting the Calot triangle in case 1. The previous biliary-intestinal anastomosis was removed in case 2.

#### Choledochoscopy

Choledochoscopy was employed through the common bile duct (CBD) in case 1 and the previous intestinal loop in case 2. No stones and strictures in the bile ducts of caudate lobe was observed in both cases and no dysfunction of Oddi sphincter in case 1.

#### Devascularization of the main liver and vasculature preservation of the caudate lobe

The hepatoduodenal ligament around porta hepatis was dissected firstly and the hepatic artery, portal vein, bile duct and their branches were exposed successively. And then the branches to caudate lobe could be preserved safely through cutting off and ligating the left and right hepatic artery, portal vein and bile duct at a safe point distal to the origin of the branches to caudate lobe (Figs. 5, 6).

#### Parenchymal transection

Liver parenchymal transection was performed with ultrasonic scalpel and cavitron ultrasonic surgical aspirator (CUSA), separating the hypertrophic caudate lobe and the atrophic main liver along their boundary. The left, middle and right hepatic veins were ligated and sutured with 5–0 Prolene.

#### Biliary drainage

T tube drainage was performed in CBD after choledochoplasty in case 1. Previous intestinal loop was used to reconstruct biliary-intestinal anastomosis in case 2.

#### Final diagnosis

Histopathological examination ruled out the potential malignancy. The final diagnosis for patient 1 was idiopathic hepatolithiasis with main liver atrophy and Spiegel lobe hypertrophy. And the diagnosis for patient 2 was hepatolithiasis secondary to previous choledochocystectomy with bilioenterostomy, leading to main liver atrophy combined with paracaval portion and caudate processus hypertrophy.

#### Outcome and follow-up

**Fig. 7:**
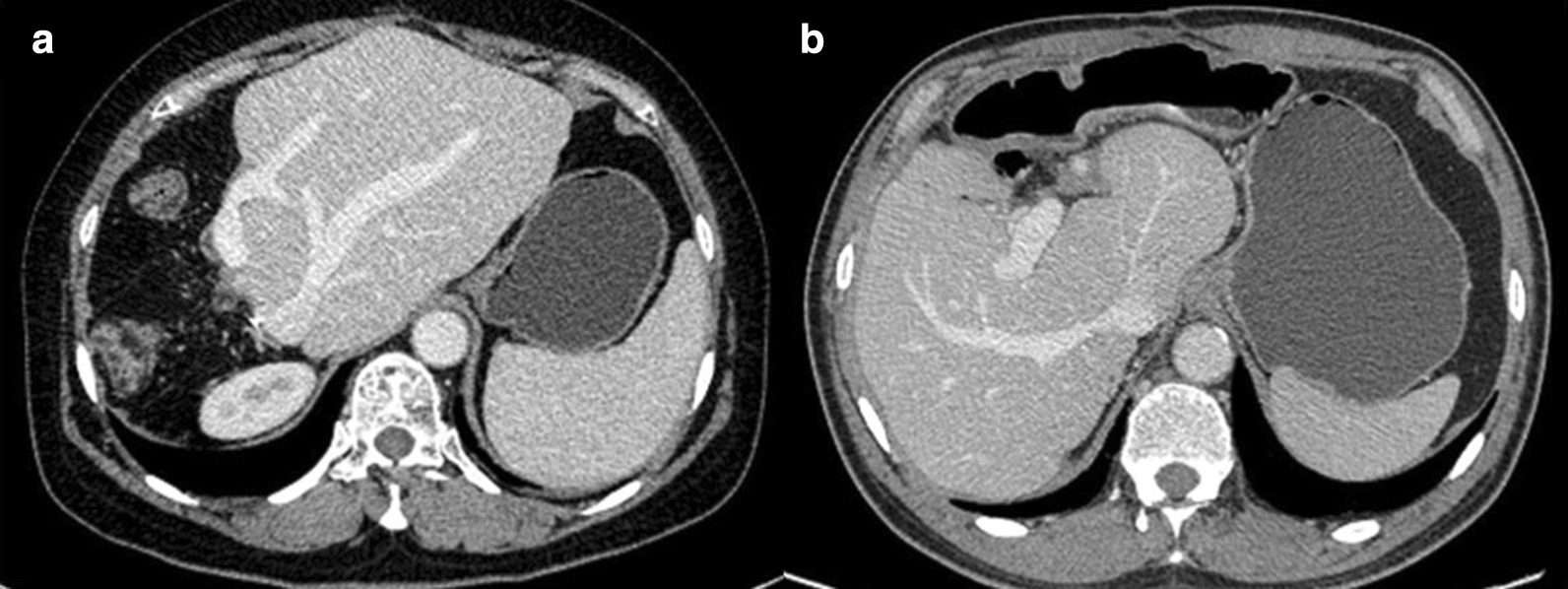
Follow-up CT image. No abnormality including stones, liver atrophy and bile duct dilatation were observed in the remanent liver of case 1 (**a**) and case 2 (**b**)

Operation time was 300 min and 360 min in two patients respectively, while blood loss was 200 ml and 300 ml. They were discharged at day 9 and 13 postoperatively without obvious complications such as bile leakage and liver dysfunction. The liver function tests, abdominal ultrasound and CT or MRI were performed at regular intervals during outpatient and telephone follow-up (Fig. 7). No evidence of liver dysfunction, hepatolithiasis recurrence or cholangitis was observed during the follow-up of 12 months in case 1 until she died of colon carcinoma. Until now, case 2 was uneventful without the above symptoms for 26 months.

## Discussion and conclusions

In 1985, Kumon firstly divided caudate lobe into three parts: Spiegel lobe, paracaval portion and caudate processus [8]. Caudate lobe is usually referred as a silent liver reserve, accounting for approximately 2% to 3% of the total liver volume (TLV) [7]. However, our study reported the extremely rare cases with utmost hypertrophy of Spiegel lobe (68.4% of TLV) in case 1 and exceeding hypertrophy of paracaval portion and caudate processus (65.8% of TLV) in case 2. The surgical outcomes of both patients demonstrate the safety and potential effectiveness of subtotal (segment II–VIII) hepatectomy. Less blood loss and shorter operation time was achieved compared with previous reports [5, 6]. Thus, our limited experience of treating bilateral diffuse hepatolithiasis with hypertrophic caudate lobe may provide a valuable reference for similar thorny cases.

Recognition of the hypertrophic caudate lobe should be the top priority during preoperative evaluation. Otherwise, misdiagnosis will be easily made, for example, the CT images of case 2 is liable to be mistaken for normal right liver and atrophic left liver with hepatolithiasis. Such inaccurate preoperative evaluations will lead to inappropriate operation planning. In that case surgeons will lose the alert to protect the blood supply and bile duct of the caudate lobe. It is practical to identify caudate lobe via thickened short hepatic veins from hypertrophic caudate lobe. On account of complete anatomic variation, it is difficult to make an intuitive judgement according to conventional CT image. In that case, employment of 3D liver reconstruction is beneficial to recognition of caudate lobe hypertrophy, assessment of liver volume and planning of the operation. Via 3D liver reconstruction, it is easy to visualize the configuration of Glisson system and hepatic veins, and to avoid intraoperative injuries to the vasculature of the caudate lobe. In that condition, dysfunction of the remnant liver or even death will occur.

As for the key points during the operation, it is difficult to distinguish the anatomic structure of hepatic hilum due to the severe adhesions and rotation of liver. We advise to apply the intrafascial approach to expose and dissect the branches of hepatic artery, portal vein and bile duct to the caudate lobe. Maneuver must be gentle and meticulous to avoid any injury to the vital blood supply or bile drainage of the caudate lobe. To differentiate the demarcation between hypertrophic caudate lobe and segment II–VIII is another critical issue. Normally, the Spiegel lobe on the left side of IVC is relatively isolated and easier to be distinguished from the peripheral liver segments, but the boundary between the paracaval portion of caudate lobe and the right posterior liver lobe is difficult to be defined. In the reports of Takayama and Midorikawa, by the means of puncturing the right posterior portal vein under the guidance of intraoperative ultrasound, the boundary between caudate lobe and right posterior liver lobe is identified by counterstaining and tattooing techniques [9, 10]. Kogure et al. have reported that the caudate processus hepatic vein can be used as the demarcation between caudate processus and right posterior lobe, but the boundary between paracaval portion and right posterior lobe is still unclear [11]. Maki et al. used 3D liver reconstruction to demonstrate that in half of normal people, a small branch from the root of right hepatic vein, named "paracaval vein", can be used as the boundary between paracaval portion and segment VII or VIII [12]. Interestingly, there is a natural demarcation between atrophic main liver and hypertrophic caudate lobe in case of AHC, which can be served as the reference line of liver parenchyma transection.

As to the choledochojejunostomy, we propose that indications should be as follows: (1) dysfunction of Oddi sphincter; (2) strictures of extrahepatic bile duct. If strictured bile duct has not been resected totally, it is easier to initiate cholangitis and recurrence of hepatolithiasis after choledochojejunostomy.

Since the bile from the caudate lobe flow upward into the hepatic duct or bilioenteric anastomosis, patients are encouraged to take intermittent prone position during sleep to promote the bile drainage and prevent cholestasis.

In conclusion, subtotal (segment II–VIII) hepatectomy under the guidance of 3D liver reconstruction is one of several treatments possible for selected patients with bilateral diffuse hepatolithiasis accompanied by compensatory caudate lobe hypertrophy. Dissecting the porta hepatis carefully and protecting the vessels and bile duct to the caudate lobe is crucial to surgical procedure.

## Supplementary information

**Additional file 1: Video 1**. Surgical video of subtotal (segment II–VIII) hepatectomy. This video shows the key steps and important anatomical structures of the surgical procedure performed on case 1.

## Data Availability

Not applicable as no dataset was used in this study.

## Abbreviations

AHC: Atrophy–hypertrophy complex
CT: Computed tomography
MRI: Magnetic resonance image
MRCP: Magnetic resonance cholangiopancreatography
RLV: Residual liver volume
CBD: Common bile duct
CUSA: Cavitron ultrasonic surgical aspirator
TLV: Total liver volume
